# Spoilage Potential of *Pseudomonas* (*P. fragi*, *P. putida*) and LAB (*Leuconostoc mesenteroides*, *Lactobacillus sakei*) Strains and Their Volatilome Profile during Storage of Sterile Pork Meat Using GC/MS and Data Analytics

**DOI:** 10.3390/foods9050633

**Published:** 2020-05-14

**Authors:** Olga S. Papadopoulou, Vasilis Iliopoulos, Athanasios Mallouchos, Efstathios Z. Panagou, Nikos Chorianopoulos, Chrysoula C. Tassou, George-John E. Nychas

**Affiliations:** 1 Laboratory of Microbiology and Biotechnology of Foods, School of Food and Nutritional Sciences, Department of Food Science and Human Nutrition, Agricultural University of Athens, Iera Odos 75, 11855 Athens, Attica, Greece; olga_papadopoulou@outlook.com (O.S.P.); heliopoylos@hotmail.com (V.I.); stathispanagou@aua.gr (E.Z.P.); 2 Hellenic Agricultural Organization “DEMETER”, Institute of Technology of Agricultural Products, Sof. Venizelou 1, 14123 Lycovrissi, Attica, Greece; nchorian@nagref.gr (N.C.); ctassou@nagref.gr (C.C.T.); 3 Laboratory of Food Chemistry and Analysis, School of Food and Nutritional Sciences, Department of Food Science and Human Nutrition, Agricultural University of Athens, Iera Odos 75, 11855 Athens, Attica, Greece

**Keywords:** aerobic storage, sterile pork meat, specific spoilage organisms, solid-phase microextraction, gas chromatography-mass spectrometry, data analytics, metabolomics

## Abstract

The aim of the present study was to investigate the evolution of the volatile compounds of aerobically stored sterile pork meat as a consequence of the metabolic activities of inoculated specific spoilage microorganisms. Thus, *Pseudomonas fragi*, *Pseudomonas putida*, *Lactobacillus sakei* and *Leuconostoc mesenteroides* were inoculated in monocultures, dual cultures and a cocktail culture of all strains on sterile pork meat stored aerobically at 4 and 10 °C. Microbiological and sensory analyses, as well as pH measurements, were performed, along with headspace solid-phase microextraction gas chromatography/mass spectroscopy (headspace SPME–GC/MS) analysis. Data analytics were used to correlate the volatile compounds with the spoilage potential of each stain using multivariate data analysis. The results for the sensory discrimination showed that the volatiles that dominated in spoiled samples consisted mostly of alcohols, ketones and two esters (butyl acetate and ethyl acetate), while at fresh samples, dimethyl sulfide, furans, acetoin and ethyl lactate were detected. On the other hand, 2-butanone, diacetyl and acetaldehyde were among the volatile compounds that were mainly correlated with the inoculated meat during storage. In addition, *P. fragi* was positively correlated with a higher number of volatiles compared to the other strains, strengthening the hypothesis that volatile compound production is strain-dependent.

## 1. Introduction

It is well-established that pseudomonads, lactic acid bacteria (LAB), *Enterobacteriaceae*, *Brochothrix thermosphacta* and clostridia are the main contributors to meat spoilage, depending on storage conditions [1,2,3,4,5]. Species of the genus pseudomonas are recognized more often as causative agents of spoilage on fresh foods of animal origin stored under aerobic conditions [6]. *Pseudomonas fluorescens,* along with the psychrotrophs *P. fragi*, *P. ludensis* and *P. putida,* are involved in the spoilage of milk, meat and fish at low storage temperatures, due to their ability to overcome the other groups of microbial consortium by converting glucose to gluconate, a substrate that can be metabolized almost exclusively by this group [7]. On the other hand, LAB and *B. thermosphacta* have been found to contribute in meat spoilage stored either under aerobic, modified atmosphere packaging (MAP) or vacuum packaging (VP). In this case, the most frequently isolated species are *Lactobacillus* spp., *Carnobacterium* spp. and *Leuconostoc* spp. [1,8]. In addition, *Lactobacillus sakei,* along with *Lactobacillus curvatus,* are considered as the predominant *Lactobacillus* species found on meat [9]. Furthermore, *Lb. sakei* and *Leuconostoc* spp. have been associated with spoilt meat stored in VP or MAP at chill temperatures [8]. In both cases, the production of metabolites depends on the dominant small fraction of the initial meat microbiota known as “specific spoilage organisms—SSO” or “ephemeral spoilage organisms—ESO” ascribing the succession of the consortia during storage [3,8,10,11]. These compounds can affect the type (e.g., saccharolytic or proteolytic) and rate of spoilage and, moreover, seem to be the principal precursors of those microbial metabolites that we perceive as spoilage [10]. Indeed, during meat spoilage, a combination of microbial and chemical activity occurs, and by-products are produced, which may be indicative for the rate and the type of spoilage [10,12]. Spoilage rate and type depends on the concentration of substrates on meat (glucose, lactic acid, nitrogenous compounds and free amino acids), which are used as principal precursors of the microbial metabolites associated with spoilage [12]. The aforementioned substrates are consumed by various microbial species, and the derived volatile fraction of the microbial metabolites consists of carbonyl compounds, alcohols, volatile fatty acids, organic acids, sulfur compounds, esters, terpenoids or other molecules that determine the spoilage of meat [4,12]. However, limited studies have been conducted to determine which bacterial species or strains are capable of producing by-products responsible for meat spoilage [4,10,13,14,15,16]. At present, it is important to study the spoilage potential at the strain level, since previous studies showed distinctions among species [13,14,17].

In the current study, sterile pork meat was stored at two different temperatures (4 and 10 °C) in aerobic conditions, after inoculation with spoilage-specific microorganisms (*Pseudomonas fragi*, *P. putida*, *Lactobacillus sakei* and *Leuconostoc mesenteroides*) in monocultures, dual cultures and a cocktail culture of the four strains. In this context, the current study was designed to monitor the different types of meat spoilage and identify the microbial metabolites produced during the metabolic activity of the strains. The overall objective of the study was the correlation of meat-volatile compounds produced by each strain with the spoilage potential of each strain. For this reason, a headspace solid-phase microextraction gas chromatography/mass spectroscopy (HS-SPME GC/MS) analysis was used for the determination of volatiles, and subsequently, a multivariate analysis (discriminant function analysis—DFA, hierarchical cluster analysis and partial least squares-discriminant analysis—PLS-DA) was performed in order to correlate them with the spoilage profile of mono, dual and mixed cultures of the selected four strains during storage at 4 and 10 °C.

## 2. Materials and Methods

### 2.1. Experimental Design and Preparation of Sterile Meat Samples

Fresh pork meat (pH 5.6–5.8) of different batches (originated from different farms) was obtained from the central meat market in Athens, Greece and transported under refrigeration to the laboratory with minimal delay (30–60 min). The raw pork meat used in the current study derived from 4 young male domestic pigs (*Sus scrofa f. domesticus*). Meat was transported to the laboratory 48 h after slaughter. In detail, 4 different batches of meat were studied, each derived from a different young male domestic pig weighing around 60–70 kg. To reduce the microbial load of the meat, the surface of the pork meat was sprayed with absolute ethanol and ignited with a gas burner. Consequently, the burnt surface tissue of the meat was removed aseptically with the aid of a sterile scalpel in a laminar flow cabinet, and the sterile tissue below was excised and cut into pieces of 45–50 g each, as described elsewhere [18]. Subsequently, the meat was inoculated with different strains of specific spoilage microorganisms, as described in Section 2.2. After inoculation, meat samples were packaged in Styrofoam trays and wrapped manually with air-permeable polyethylene plastic film, ensuring that there was no direct contact of the plastic film with the surface of the meat. All samples were stored at 4 and 10 °C in high-precision (±0.5 °C) incubation chambers (MIR-153, Sanyo Electric Co., Osaka, Japan) for up to 187 h depending on storage temperature, until spoilage was very pronounced (intense discoloration and presence of off-odors). Sampling was performed every 8 h and 12 h for meat stored at 4 °C and 10 °C, respectively. Each sample was divided into two portions of 25 g. One portion was used for microbiological analysis and the other one for GC/MS analysis. At each time point, two packages were withdrawn randomly and analyzed for each storage condition to undergo microbiological and GC/MS analysis, and two packages were employed in sensory evaluation. Overall, 480 samples were analyzed for microbiological, chemical analysis and sensory assessment.

### 2.2. Preparation of Inoculum and Inoculation of the Sterile Pork Meat

The microorganisms used for the inoculation of sterile meat were: *Pseudomonas fragi* (CF43, isolated from beef fillet during storage at 0 °C, accession number HQ014889), *Pseudomonas putida* (CF7, isolated from beef fillet during storage at 20 °C, accession number HQ014881), *Lactobacillus sakei* (MF41, isolated from beef fillet during storage at 0 °C, accession number HQ014896) and *Leuconostoc mesenteroides* (MF5, isolated from beef fillet during storage at 20 °C, accession number HQ014894). All the strains were activated from a stock culture stored at −80 °C and were subcultured in 10-mL brain heart infusion broth (BHI; LAB049, LabM, Lancashire, UK) for *Pseudomonas* spp. and De Man-Rogosa-Sharpe (MRS) broth (094, LabM) for lactic acid bacteria (LAB) incubated at 25 and 30 °C for 24 h, respectively. The cells were harvested by centrifugation (5000× *g*, 10 min, 4 °C), washed twice with 10-mL quarter-strength Ringer’s solution (Ringer’s solution tablets, 96724-100TAB, Merck, Darmstadt, Germany) and resuspended in 10 mL of the same medium. The concentration of the individual cultures was approximately 8.5–9.0 log CFU/mL, as assessed by pour-plating on MRS agar (MRS ISO; 4017282, Biolife, Milano, Italy) for LAB and spread-plating on Pseudomonas agar base (PAB; CM559 supplemented with Centrimide- Fucidin- Cefalosporin selective supplement CFC SR0103, Oxoid, Basingstoke, UK) for *Pseudomonas* spp. All strains were then serially diluted with Ringer’s solution to give a final concentration of approximately 2 log CFU/g in the surface of the pork fillet. For the preparation of the dual cultures and the cocktail culture of the 4 strains, the monocultures were mixed in equal volumes. Pork fillets were inoculated with monocultures, dual cultures and a cocktail culture of 4 strains (Table S1). Sterile uninoculated meat samples were also prepared and used as control.

### 2.3. Microbiological Analyses

Microbiological analyses were carried out until the end of storage at 4 and 10 °C. To estimate the number of viable cells, 25 g of pork meat were aseptically added to 50-mL sterile one-quarter strength Ringer’s solution (to reduce the detection limit of the enumeration method to 1.48 log CFU/g) and homogenized in a stomacher device (Stomacher 400 circulator, Seward Limited, Norfolk, UK) for 60 s at room temperature. The resulting suspensions were serially diluted in the same diluent and 1 or 0.1-mL samples were poured or spread in triplicate on the following agar media: plate count agar (PCA; 4021452, Biolife) for the enumeration of total viable counts (TVC), incubated at 30 °C for 48–72 h; Pseudomonas agar base with CFC-selective supplement (Oxoid) for the enumeration of *Pseudomonas* spp., incubated at 25 °C for 48–72 h, and MRS agar (Biolife) overlaid with the same medium for the enumeration of LAB, incubated at 30 °C for 48–72 h. In addition, from the resulting suspensions, 1 mL of the first decimal dilution was equally spread on 3 agar plates of each substrate (for spread plating) to ensure that the microbiota was lower than 0.48 log CFU/g during storage of the sterile meat.

In addition to the microbiological analysis, the pH values of the pork meat were also recorded with a digital pH meter (Metrohm pH Lab, Herisau, Switzerland) by immersing the glass electrode in the meat homogenate (stomacher homogenate) after the end of the microbiological analysis.

### 2.4. Sensory Analysis

The sensory assessment of the pork samples was performed throughout storage as reported elsewhere [19] by a seven-member trained staff from the laboratory, at the same time intervals as for microbiological analyses. In brief, the sensory panel evaluated color, smell and taste (after cooking) of the meat using a three-class evaluation scheme. The first class (fresh) corresponded to acceptable meat quality and the absence of off-flavors (scores 1–2), the second class (semi-fresh) corresponded to the presence of slight off-flavors but not spoiled (still acceptable quality) (scores 3–5) and the third class (spoiled) corresponded to clearly off-flavor development (unacceptable quality) (scores 5–10). Scores ≥5 indicated the end of shelf-life. Semi-fresh was the first indication of meat spoilage (incipient spoilage, i.e., less vivid red color, odor and flavor slightly changed, but still acceptable by the consumer) in which the sample was marginally accepted.

### 2.5. Determination of Volatile Compounds by Headspace SPME-GC/MS

Headspace SPME–GC/MS analysis was used for the analysis of the volatile compounds of pork meat, as reported elsewhere [20], with slight modifications. In brief, 2.5 g of pork fillet, 5 mL of 25% (*w*/*v*) NaCl solution and 10 μL of internal standard (4-methyl-1-pentanol, in vial concentration 1000 μg/L) were homogenized with a glass rod for 2 min into a 20-mL glass vial. The vial was hermetically closed with a Mininert valve (Sigma Aldrich, St. Louis, MO, USA), and the content was stirred at 40 °C for 15 min. Subsequently, the SPME fiber - Divinylbenzene/Carboxen/Polydimethylsiloxane (DVB/CAR/PDMS—50/30 μm, needle length 1 cm, needle size 24 ga; Sigma Aldrich) was inserted to the glass vial and exposed to the headspace for 30 min under the same conditions. Desorption of the volatiles took place in the injector of the GC/MS for 5 min. Before each analysis, the fiber was exposed to the injection port of another GC (270 °C) for 10 min to remove any volatile contaminants. GC/MS analyses were performed on an Agilent 7890A gas chromatograph coupled to an Agilent 5973C mass spectrometer (Agilent Technologies, Santa Clara, CA, USA). The injection port was equipped with a liner (0.75-mm i.d.) suitable for SPME analysis. It was operated in split mode (split ratio 2:1) at 250 °C. The gas chromatograph was equipped with an HP-5MS column (30 m, 0.25-mm i.d., 0.25-μm film thickness; Agilent Technologies), and the carrier gas was helium at a constant flow rate (0.93 mL/min). For the analysis of the volatile compounds, oven temperature was programmed from 40 °C (held for 5 min) to 150 °C at a rate of 4 °C/min and then ramped to 250 °C at a rate of 30 °C/min; it was held there for 5 min. The temperature of the interface, MS source and quadrupole were set at 280, 230 and 150 °C, respectively. The mass spectrometer was operated in electron ionization mode with the electron energy set at 70 eV and mass scan range 29-350 Da (scan rate: 4.37 scans/s, gain factor: 1, resulting EM voltage: 1188 V).

The compounds were identified by comparing: (i) the retention indices (RI) based on a homologous series of even-numbered n-alkanes (C8–C24; Niles, IL, USA) with those of standard compounds and by comparison with the literature data and (ii) MS data with those of the reference compounds and by MS data obtained from the National Institute of Standards and Technology (NIST) library (NIST/EPA/NIH Mass Spectral Library with Search Program, data version NIST 05, software version 2.0d) and Wiley Registry of Mass Spectral Data 7th edition. Amdis software (version 2.62, http://chemdata.nist.gov/dokuwiki/doku.php?id=chemdata:amdis) was used for the deconvolution of mass spectra and identification of target components.

The volatile compounds were semi-quantified by dividing the peak areas of the compounds of interest by the peak area of the internal standard (IS) and multiplying this ratio by the concentration of the IS (expressed as μg/L). The peak areas were measured by selecting single ions.

In total, 208 samples were analyzed for both temperatures. The samples for the volatile compound analysis were selected in order to cover a wide range of samples representative for the inoculated meat with the specific spoilage microorganism cases and the sterile (control) pork meat for both storage conditions, according to the microbial counts and the sensory scores.

### 2.6. Estimation of Growth Kinetic Parameters and Data Analysis

The primary model of Baranyi and Roberts [21] was applied to the growth data from plate counts (log transformed) using the online program DMFit (available at www.combase.cc) to determine the kinetic parameters of microbial growth, namely the maximum specific growth rate (*μ_max_*) and the lag phase duration (λ).

Data mining and interpretation were performed using multivariate statistical methods. The dataset was divided in groups depending on (i) the different strains used for the inoculation of sterile pork meat and ii) the different sensory classes (F, SF and S). The applied data matrix contained the volatile compounds (X variables) and the meat samples (Y variables). Data were transformed by scaling (autoscale) as a column-wise normalization step in order to make each variable comparable to each other [22]. The formed data matrix was uploaded in the online platform “MetaboAnalyst v3.0” (McGill University-Xia Lab, Montreal, QC, Canada), which is a web-based metabolomic data-processing tool (www.metaboanalyst.ca) [23]. Data analytics (partial least squares-discriminant analysis (PLS-DA)), hierarchical cluster analysis (dendrogram and heatmaps) and univariate analysis (one-way analysis of variance (one-way ANOVA) and *t*-test) were performed in the applied datasets.

Additionally, discriminant function analysis (DFA) was applied using the XLStat software ver. 2006.06 (Addinsoft, Paris, France), a built-in statistical software package of Excel. DFA is a supervised classification method, in which the decision boundary between different classes is calculated so that the variance between the classes is maximized and the variance within the individual classes is minimized [24]. DFA was used to explore the relationship between variables (volatile compounds) and sterile and inoculated pork samples during storage and discriminate the meat samples in the predefined classes. Data were randomly diversified into train (65 samples) and test data (36 samples). The classification accuracy was determined by the number of classified samples in each class divided by the total number of samples in the class.

## 3. Results and Discussion

### 3.1. Development of Microbial Association and Sensory Analysis

**Figure 1:**
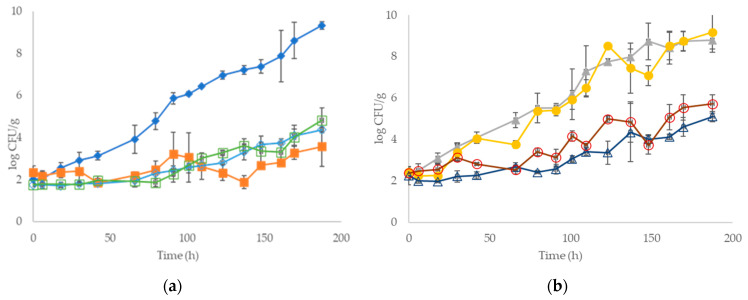

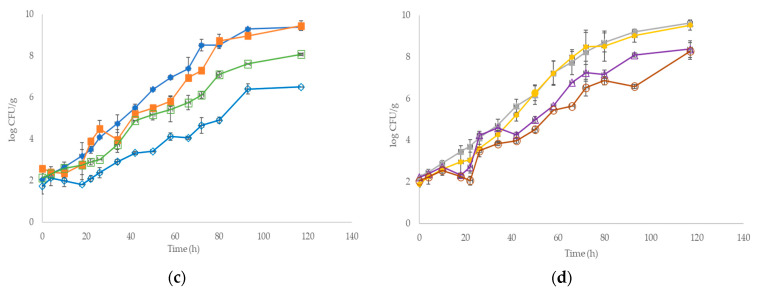
Population dynamics of the inoculated strains during the storage of pork fillets at 4 °C (**a**) and (**b**) and at 10 °C (**c**) and (**d**). (♦): *Pseudomonas fragi*, (■): *P. putida*, (▲): co-culture of *P. fragi* and *P. putida*, (●): population of *P. fragi* and *P. putida* in the cocktail culture of the four strains, (◊): *Lactobacillus sakei,* (□) *Leuconostoc mesenteroides*, (**∆**): co-culture of *Lb. sakei* and *Ln. mesenteroides* and (**◯**): population of *Lb. sakei* and *Ln. mesenteroides* in the cocktail culture of the 4 strains. Datapoints represent mean values ± standard deviation of two replicated samples.

The population dynamics of the inoculated pork fillet samples with different strains during storage at 4 and 10 °C is presented in Figure 1. The microbiota after the inoculation of sterile pork fillets was approximately 2 log CFU/g and consisted of monocultures, dual cultures and a cocktail culture of the four strains of *P. fragi*, *P. putida*, *Lb. sakei* and *Ln. mesenteroides*, depending on each case (Table S1). According to the results, the population of all microorganisms in the pork samples increased during storage at both storage temperatures.

In detail, regarding the pork samples inoculated with monocultures of the strains, results showed that at 4 °C *P. fragi* reached the highest population level at the end of storage (9.33 ± 0.18 log CFU/g), while the lag phase of this strain was the shortest compared to the other monocultures (Table 1). *Ln. mesenteroides* followed with a final population level of approximately 4.82 ± 0.59 log CFU/g, while the population levels of *P. putida* and *Lb. sakei* were found lower and with similar counts (3.56 ± 0.93 and 4.37 ± 0.10 log CFU/g, respectively). Accordingly, for the inoculated meat samples with dual and cocktail cultures at the same temperature, the population of *P. fragi* and *P. putida* was ≥ 8.79 log CFU/g in all cases (8.79 ± 0.42 and 9.13 ± 0.97 log CFU/g for dual and cocktail cultures, respectively), while the LAB populations remained at lower levels (5.10 ± 0.25 and 5.72 ± 0.43 log CFU/g for the dual and cocktail culture, respectively).

In storage at 10 °C, all samples exhibited similar or higher population levels compared to 4 °C (Figure 1c,d). Specifically, *P. fragi* and *P. putida* exceeded 9.0 log CFU/g in all cases, whereas, for the samples inoculated with the monoculture of *Ln. mesenteroides,* dual and cocktail cultures of LAB, population levels exceeded 8.0 log CFU/g (8.06 ± 0.05, 8.37 ± 0.40 and 8.27 ± 0.40 log CFU/g, respectively) (Figure 1c,d). On the contrary, the inoculated meat with the monoculture of *Lb. sakei* displayed the lowest population (6.50 log CFU/g) during storage at 10 °C.

The changes in the population of these groups and their contribution to the final microbiota were influenced by storage temperature, as illustrated by the population dynamics on the specific growth media. Growth kinetic parameters, such as lag phase duration (*λ*) and maximum specific growth rate (*μ_max_*) of the different inoculation treatments, are presented in Table 1; Table 2. In general, during cold storage, the growth rate of *P. putida* was lower compared to *P. fragi* (Table 1), which could be attributed to the fact that the former strain had been previously isolated from beef during storage at 20 °C, whereas the latter (*P. fragi*) was isolated from beef at refrigerated storage (0 °C). The same holds for *Ln. mesenteroides,* which was isolated from beef during storage at 20 °C. However, despite the fact that *Lb. sakei* was also isolated from beef during storage at 0 °C and its potential psychrotrophic nature [8], it was not able to grow well at 4 °C as a monoculture.

Prolonged lag phases were evident for inoculated meat with LAB, as well as for inoculated meat with a monoculture of *P. putida* at 4 °C, the duration of which was greatly reduced at the higher storage temperature in all cases (Table 1; Table 2). Additionally, a progressive increase of the maximum specific growth rate (*μ_max_*) was observed for all inoculated strains with increasing storage temperature. In general, aerobic storage at 10 °C accelerated spoilage of the samples inoculated with dual culture of *P. fragi* and *P. putida* and the cocktail culture of the four strains, due to the fast growth of *P. fragi* and *P. putida* that became the dominant species. Finally, it has to be noted that no microorganisms were found during storage of the sterile meat samples at both temperature conditions.

Ercolini et al. [13] used three bacterial strains, namely *P. fragi*, *Serratia proteamaculans* and *Carnobacterium maltaromaticum,* as single or mixed cultures to inoculate sterile beef packaged under vacuum and stored at 7 °C for a month. It was reported that the populations of the inoculated samples increased during storage for all strains used compared with uninoculated meat, where lower counts were found. In detail, *Carnobacterium maltaromaticum* showed the highest increase when it was inoculated as a single culture, whereas *P. fragi* displayed the highest increase in the inoculated meat when applied in a mixed culture. In a similar study [14], beef meat was inoculated with 18 strains of *P. fragi* as a monoculture (4.0 log CFU/g initial inoculum level) and stored at 4 °C for seven days. After one week of storage, the load of the inoculated strains increased to 9.0 log CFU/g, indicating that *P. fragi* had a significant role in the microbial ecology of meat, and thus, meat could be considered an ecological niche for this species.

The pH values of fresh pork were found within the normal range of fresh pork (pH 5.8 ± 0.4). During the aerobic storage of meat, the pH values fluctuated (prior to spoilage) within the normal pH range of fresh meat. When meat was characterized as spoilt, pH increased or decreased depending on the inoculation treatment. More specifically, pH increased in all spoiled samples inoculated with *P. fragi* and *P. putida* (mono cultures, dual cultures and in mixed strain cultures) (*p* < 0.05), where the highest increase was observed in the case of meat inoculated with a monoculture of *P. fragi* at both temperatures (4 and 10 °C), reaching values of 6.5–6.8. However, for samples inoculated with LAB (mono and dual cultures), a slight pH decrease was evident in spoiled meat, depending on storage temperature. At 10 °C, pH of spoiled meat was found slightly lower (pH 5.6 ± 0.3) in contrast to cold storage, where the pH values were approximately 5.7 ± 0.2. Those observations are in-line with previous works in aerobic stored meat when *Pseudomonas* spp. become the dominant microbiota, and it is attributed to the by-products produced, which can increase the pH values of the meat [10,20]. On the other hand, LAB are able to produce lactic acid during the spoilage of meat and, thus, to lower the pH of the meat [10].

According to the sensory analysis (Table S2), the shelf life of pork fillets differed depending on storage temperature and the specific microorganism used for inoculation. More specifically, the sensory panel considered a sample as spoiled in a shorter time when inoculated with *P. fragi* than with *P. putida*. In detail, meat inoculated with *P. fragi* or the mixed species culture were characterized as semi-fresh after 54-h storage at 4 °C, while for *P. putida* and the dual culture of *P. fragi* and *P. putida*, the corresponding time was 68 h at the same storage temperature. The time of sensory rejection (meat characterized as spoiled) was 116 h when the meat was inoculated with *P. fragi,* and the population level was 7.09 log CFU/g, while for the dual culture of *P. fragi* and *P. putida* or the mixed species cocktail, the corresponding time was 106 h, and the final population was 6.70 log CFU/g. In this case, the odor of the meat was intense, and slime had formed on the surface of the fillets. This coincides with the glucose depletion, where a number of genes, e.g., PP5337, Asp, purE and aruF, are overexpressed and have been correlated with spoilage [25].

In contrast for meat inoculated with LAB strains, sensory rejection was reported by the panel after 124.5 and 140 h for mono and dual LAB cultures, respectively, indicating that spoilage from LAB is milder and mostly characterized by muscle souring [10,26]. At 10 °C, the time needed for sample rejection was reduced compared to 4 °C, while the population of the microorganisms was higher (Table S2).

In a recent study [14], 18 strains of *P. fragi* were used as inocula to spoil meat during aerobic storage at 4 °C. In this work, a sensory panel assessed the different meat odors present in the samples during storage, and the results showed that the different strains affected significantly the meat odor. Thus, it was concluded that the sensory profiles of inoculated meat were strain-dependent.

### 3.2. Discrimination of Sterile From Inoculated Meat Using Volatile Compounds

**Figure 2:**
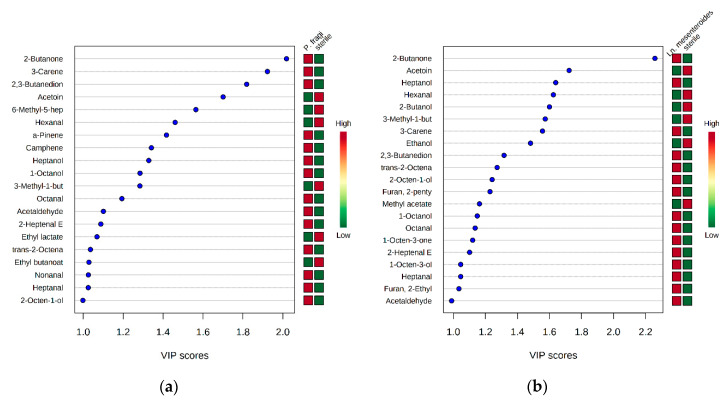
Important features identified by partial least squares-discriminant analysis (PLS-DA) for sterile meat and meat inoculated with: (**a**) *P. fragi* (monoculture) and (**b**) *Ln. mesenteroides* (monoculture) stored at 4 and 10 °C. The color scale on the right represents the scaled abundance of each variable, with red color boxes indicating high abundance and green color boxes indicating low abundance.

Fifty-one (51) volatile compounds were detected during the storage of pork fillets at both temperatures. The majority of them consisted of alcohols, aldehydes, ketones, esters, terpenoids and sulfur compounds. Table S3 presents the 51 volatile compounds detected for all meat cases (sterile and inoculated). Accordingly, the 51 volatile compounds were selected and used for quantitative estimation and discrimination of the meat samples based on the different inocula employed, as well as in sensory classification. Many of the selected compounds, such as 1-octen-3-ol, diacetyl, acetoin, 2-butanone and ethyl acetate, have already been identified to be involved in meat spoilage [4,12,20,27,28]. The selected data were uploaded to the online platform MetaboAnalyst v3.0 (www.metaboanalyst.ca). Significant differences between the sterile meat and each case of monocultures of inoculated meat were observed. Compounds with significant effects (*p* < 0.05) during aerobic storage after *t*-test analysis were found to be 2-butanone, acetoin, diacetyl, hexanal, 6-methyl-5-hepten-2-one, 3-methyl-1-butanol, heptanol, 2-butanol, ethanol and 3-carene (data not shown). Subsequently, PLS-DA was applied in order to extract the important features (volatile compounds) for each case of monocultures and dual cultures in relation to the sterile meat. PLS-DA analysis has some advantages, like discriminating samples between known classes and/or predicting unknown samples, but also, it can associate metabolite data with each class [29]. Regarding the results obtained from the treatment of sterile meat versus inoculated meat with *P. fragi*, it was shown that most of the compounds were highly correlated with the inoculated meat (Figure 2a).

Specifically, *P. fragi* was positively correlated with 2-butanone, 3-carene, diacetyl and acetaldehyde, while acetoin and hexanal were correlated with sterile meat (Figure 2a). As regards *Ln. mesenteroides*, PLS-DA showed that the inoculated meat was mostly correlated with 2-butanone, diacetyl, alcohols and carbonyls with seven or eight carbon atoms, 2-pentyl-furan and 2-ethyl-furan, while sterile meat was positively correlated with acetoin, hexanal, 2-butanol and 3-methyl-1-butanol (Figure 2b). At the other two inoculation cases with *P. putida* and *Lb. sakei*, 2-butanone had the highest VIP score (≥2.2) for the inoculated meat (Figure S1).

According to hierarchical cluster analysis, a clear discrimination between the sterile meat and that inoculated with *P. fragi* and *P. putida* (mono and dual cultures) stored at 4 and 10 °C was shown. One major group corresponded to the inoculated meat with *P. fragi* and *P. putida* stored at 10 °C, and a second major group was divided into two subgroups: one relevant to sterile meat and another relevant to samples inoculated with *P. fragi* and *P. putida* (including all samples stored at 4 °C and two of the samples stored at 10 °C) (Figure S2).

**Figure 3:**
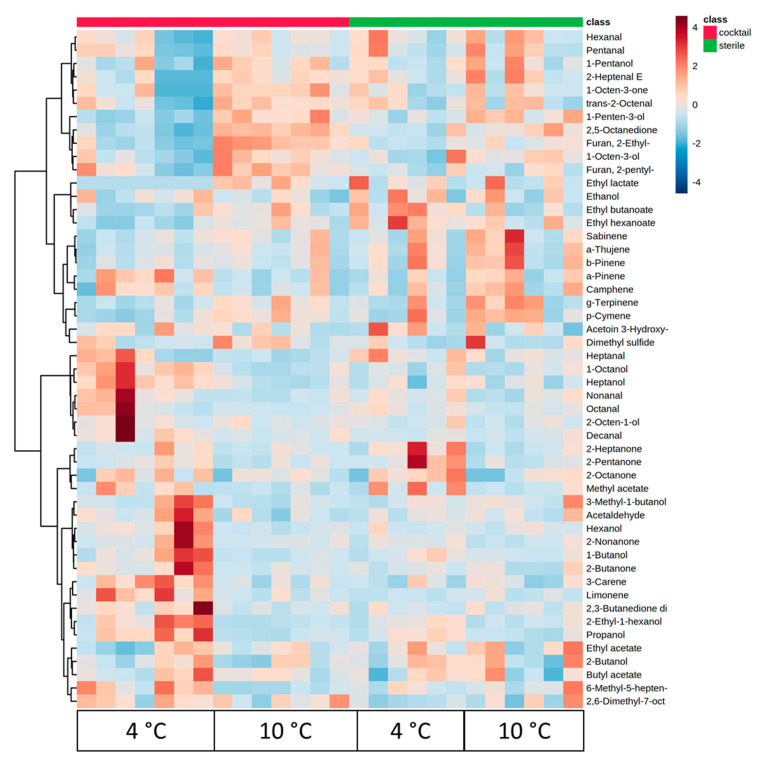
Hierarchical clustering result shown as a heatmap of volatiles associated with the group of sterile meat (green) and meat inoculated with the cocktail of all cultures (red) during storage at 4 and 10 °C. Ward-linkage clustering was based on the Euclidean correlation coefficients of the 51 volatiles identified in the different meat samples. The color scale represents the scaled abundance of each variable, with red indicating high abundance and blue indicating low abundance.

Figure 3 presents the heatmap obtained from the treatment of a combination sterile meat versus meat inoculated with all strains. It can be observed that inoculated meat at 4 °C differentiated from the other cases, and the volatiles positively correlated with that included mainly alcohols, acetaldehyde diacetyl, 2-nonanone, butyl and ethyl acetate and 2 monoterpenes.

**Figure 4:**
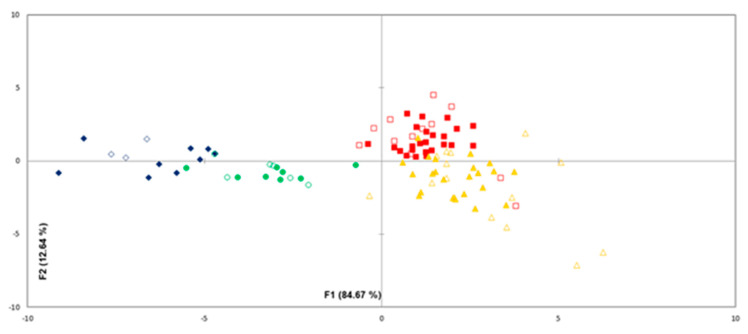
Discriminant analysis similarity map for the different microbial groups (♦): sterile meat, (▲): meat inoculated with microbial species of pseudomonads, (■): meat inoculated with microbial species of LAB and (●): meat inoculated with a cocktail culture of 4 strains stored at 4 and 10 °C, as determined by the first two discriminant functions. Empty and filled symbols indicate the validation and training samples, respectively.

Discriminant function analysis (DFA) was used in order to discriminate meat samples based on the different inocula. As illustrated in Figure 4, when data were combined in a single dataset, sterile meat samples, cultures of *P. fragi* and *P. putida* (monocultures and dual cultures), cultures of LAB (monocultures and dual cultures) and mixed cultures (four strain cultures) constituted four separate clusters (Figure 4). However, 15% of the samples were misclassified. The correct classification rate for the validation of the DFA model was 77.0% for the LAB samples, 71.4% for the *P. fragi* and *P. putida* samples, 83.3% for the mixed cultures and 100% for the sterile meat samples, with an overall accuracy performance of 77.8%. These results revealed a satisfactory correlation between the inoculated meat samples and the sterile meat, along with the dynamic changes of the amounts of the volatile compounds. As it is evident, sterile meat and inoculated meat treatments formed separate clusters, indicating that data analytics in tandem with GC/MS could determine differences between the volatile compounds produced by the various microorganisms used in the current study.

According to the previous results, 2-butanone, diacetyl and acetaldehyde were among the volatile compounds that were mainly correlated with the inoculated meat. Previous studies have shown that diacetyl and acetaldehyde could derive from acetoin breakdown through an acetoin dehydrogenase enzyme system, a mechanism that works widely in bacteria, according to the review of Xiao and Xu [30]. Huang et al. [31] recognized that *P. putida* belongs to the acetoin-utilizing microorganisms. In detail, 2,3-butanediol is metabolized by oxidation to acetoin, and then, acetoin is metabolized to acetaldehyde and acetyl-CoA by the acetoin-cleaving system [31]. In the current study, 2,3-butanediol was not detected in every case of both sterile and inoculated meat. However, acetoin, which was detected in all samples, decreased during storage of the inoculated samples only, and this was more evident in the samples inoculated with *P. fragi*. Acetoin concentration was the highest in the case of sterile meat and presented a small increase in meat inoculated with *P. putida* and with the cocktail cultures of four strains, while it presented a decreasing trend in the inoculated meat with *Ln. mesenteroides*, dual species of pseudomonads and LAB at 4 °C. Regarding the inoculated meat with the monocultures of *P. fragi* and *Lb. sakei*, acetoin was found in low levels during storage at 4 °C and 10 °C. Yet, it has to be pointed out that an acetoin concentration was found in all cases of inoculated meat at very low levels in comparison with the sterile meat, where its concentration was tenfold higher. As regards diacetyl, it increased or fluctuated in the cases of inoculated meat, whereas in sterile meat, it was detected at low levels at both temperatures during storage. On the other hand, hexanal showed the highest concentration in all samples in the beginning of storage, and this could be attributed to the oxidation of oleic and arachidonic acids usually found on pork meat [13,32]. Nurjuliana et al. [33] observed that 2-butanone, acetoin and diacetyl were among the main volatiles of fresh pork meat. Ferrocino et al. [27] reported that acetoin was produced in the highest quantity under a vacuum storage of meat at 1 °C [27], while Casaburi et al. [17] observed that the aforenoted compound was one of the most frequent volatiles found in the headspace of meat samples stored under aerobic and vacuum packaged. It is well-documented that ketones derive from the oxidation of fatty acids due to chemical autoxidation or enzymatic *a-* and *b*-oxidation [13,34] or are associated with the presence of Gram-negative (i.e., *Pseudomonas, Shewanella* and *Moraxella*) or Gram-positive bacteria (*Carnobacterium* spp.), which form ketones from other metabolic pathways, such as alkane degradation or dehydrogenation of alcohols [4,6,13]. Casaburi et al. [12] reported that *P. fragi* and *P. fluorescence* were responsible for the production of a great number of aldehydes, such as 2-methyl-butanal, 3-methyl-butanal, hexanal, heptanal, octanal, nonanal and decanal, some of which were also identified in the present study. However, Casaburi et al. [17] and Ercolini et al. [4,14] reported that, among the carbonyl compounds, nonanal and acetoin could be used as meat-spoilage tracers, since they are often found in spoilt meat, results that are not in-line with the current study.

### 3.3. Correlation of Meat Sensory Classes with Volatile Compounds

**Figure 5:**
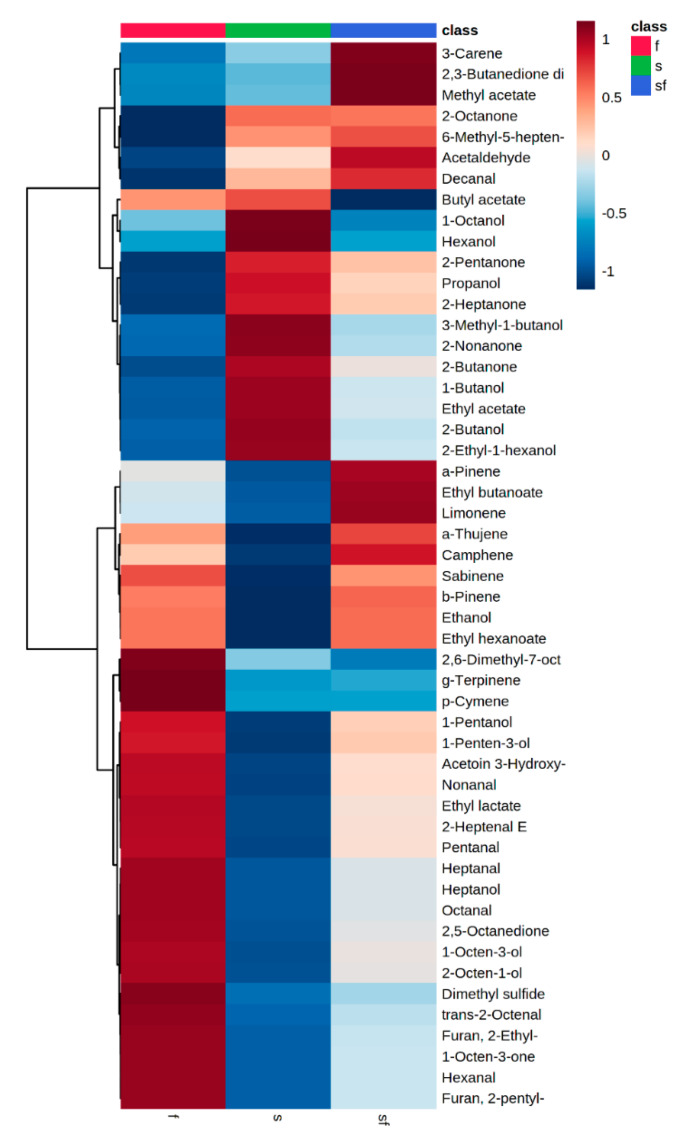
Heatmap of the volatiles clustered into 3 sensory classes of the sterile and inoculated meat samples during storage at 4 and 10 °C. Ward-linkage clustering was based on the Euclidean correlation coefficients of the 51 volatiles identified in the different sensory classes. The color scale represents the scaled abundance of each variable, with red indicating high abundance and blue indicating low abundance. Columns are colored according to the sensory class: fresh (red), semi-fresh (blue) and spoiled (green).

In the case of discrimination between the sensory classes, the selected data (51 volatile compounds) were uploaded to the online platform MetaboAnalyst v3.0 (www.metaboanalyst.ca). From one-way ANOVA, 20 compounds were found significant (*p* < 0.05) during aerobic storage (data not shown). The differences in the volatile compounds between the sensory classes of aerobically stored meat are clearly distinct in the clustering result shown as a heatmap (Figure 5).

The volatile compounds of fresh samples were dominated by three terpenoids, dimethyl sulfide, furans, eight aldehydes, alcohols with seven or eight carbon atoms, unsaturated ketones with eight carbon atoms, acetoin and ethyl lactate. Subsequently, semi-fresh samples were correlated with seven terpenoids, ethanol, acetaldehyde, decanal, diacetyl, methyl acetate, ethyl butanoate and ethyl hexanoate. Finally, the volatiles that dominated in spoiled samples consisted mostly of alcohols, ketones and two esters (butyl acetate and ethyl acetate).

According to Figure 5, fresh samples were correlated with alcohols such as 1-pentanol, 1-penten-3-ol, 1-octen-3-ol, and 2-octen-1-ol, whereas 2-ethyl-1-hexanol was positively correlated with spoiled samples. Some of the identified alcohols of this study are commonly found in fresh meat during storage in aerobic conditions, according to a recent review [1]. Insausti et al. [32] reported that, in beef meat stored under MAP, four alcohols were detected: ethanol, 1-pentanol, 1-penten-3-ol and 1-octen-3-ol and contributed to the butter, sweet, acidic or mushroom odors [32]. In other studies, efforts have been made to correlate volatiles as spoilage indicators. In that sense, Ferrocino et al. [27] characterized two alcohols, i.e., 2-ethyl-1-hexanol and 1-octen-3-ol, as possible meat-spoilage indicators. The former compound (2-ethyl-1-hexanol) is related to resin, flowers and green odors [1], while the latter (1-octen-3-ol) is a key odorant in cheeses [17]. On the contrary, in the work of Jääskeläinen et al. [35], where meat was stored under high-oxygen MAP packaging, it was observed that 1-octen-3-ol originated from chemical reactions rather than bacterial metabolic activity. However, in the current study, the production of different alcohols seemed to be strain-dependent. Alcohol production could be attributed to many metabolic pathways, where Gram-negative bacteria such as *Pseudomonas* spp. and Gram-positive bacteria such as *Carnobacterium* spp. play a major role [12]. Glucose catabolism, proteolytic activity, amino acid metabolism, oxidation of fatty acids and methyl ketone reduction, along with the reduction of aldehydes, are the main metabolic pathways involved in the biosynthesis of volatile alcohols [10,12,25].

Among the ketones detected, 2-butanone, 2-pentanone, 2-heptanone and 2-nonanone were correlated with spoiled samples, while acetoin was correlated with the fresh samples (Figure 5). On the other hand, aldehydes such pentanal, hexanal, heptanal, octanal, *trans*-2-octenal and nonanal were associated mostly with fresh samples. Argyri et al. (2015) reported that, when minced beef was stored under aerobic and MAP conditions, 2-pentanone, 2-heptanone, 2-octanone, 3-octanone, acetoin and diacetyl increased at both storage conditions, while 2-butanone decreased during the storage of minced beef. Moreover, these researchers observed an association between fresh and semi-fresh minced beef samples stored under different packaging conditions with the volatile compounds 2-butanone, 2,3-pentanedione, 2,5-octanedione, pentanal, hexanal, *trans*-2-heptanal and *trans*-2-octenal, while the compounds positively correlated with the spoiled samples were found to be 2-pentanone, 2-nonanone, 2-and 3-methyl-1-butanol, ethyl hexanoate, ethyl propanoate, ethyl lactate, ethyl acetate, ethanol, 2-heptanone, 3-octanone, diacetyl and acetoin. Many of the volatile compounds reported by Argyri et al. [20] were not found in accordance with the current study; however, their study differentiated from the present work in terms of meat type, storage temperatures and packaging, factors which could affect the produced volatiles. In air-packaged beef inoculated with strains of *P. fragi*, the most common detected molecules were ethyl hexanoate, ethyl octanoate and ethyl decanoate, and according to the sensory analysis, fruity off-odors of meat were associated with the ester release [14]. Those results are in-line with the present study, since the detected esters were mostly associated with the semi-fresh and spoiled samples.

Saraiva et al. [36] used data analytics in tandem with GC/MS to indicate the volatile compounds as beef spoilage indicators in meat stored under MAP and vacuum-packaged at 4 °C. They demonstrated that compounds such as 2- and 3-methylbutanal, 2- and 3-methylbutanol, 1-pentanol, 1-hexanol, 2,3-octanedione, 3,5-octanedione, octanal and nonanal can be used as meat-spoilage tracers, while 3-methylpentane can be used as an indicator of the early stages of beef spoilage. Accordingly, Insausti et al. [32] discriminated meat quality in terms of storage time and observed that dimethyl sulfide was correlated with fresh meat, whereas acetone and ethanol were associated with meat stored under MAP for longer periods. Resconi et al. [37] proposed pentanoic, hexanoic and heptanoic acid, 1-hexanol, 2-undecenal, ethyl octanoate, 2-heptanone and 2-pentylfuran as shelf-life markers of raw beef in high-oxygen MAP. The concentration of esters in all the inoculated cases started to decline after 50 h of storage until the end of aerobic storage. Insausti et al. [32] reported that esters derive from the esterification of several alcohols with carboxylic acids present on bovine meat. In addition, Ercolini et al. [13] concluded that microbial esterases are responsible for the formation of ethyl esters due to the esterification of alcohols with carboxylic acids. In another study [6], *Pseudomonas, Shewanella* and *Moraxella* were found capable of producing methyl- and ethyl-esters. Finally, the monoterpenes 3-carene, a-pinene, limonene and camphene, which were mostly correlated with the inoculated cases of meat, are known to derive from bacteria [38]. It is obvious that contradictory results are found in the literature as regards the volatiles identified in meat during storage. However, the majority of the aforenoted studies dealt with meat stored under MAP and/or vacuum packages, a factor that is known to affect the microbial association on the type of the produced metabolites and the type of spoilage [10].

The results obtained in the present study demonstrated that different microbial groups and/or different strains resulted in different volatile profiles during spoilage. The latter could be considered as fingerprints containing valuable information for each case of inoculated meat. Compounds such as hexanal, 2-butanone, diacetyl and/or acetoin were found to be statistically significant in all cases examined. However, hexanal is known to be produced from the oxidation of unsaturated ω-6 fatty acids or the degradation of amino acids [13,39]. Accordingly, hexanal was found in both sterile and inoculated meat, and therefore, it cannot be used as an indicator of microbial spoilage, since the concentration of this compound depended also on nonmicrobial factors. *Pseudomonas* are capable of metabolizing acetoin, whereas LAB are capable of producing it [1,31,40]. However, in the present study, acetoin and diacetyl were found in low levels throughout the storage of meat inoculated with a monoculture of *Lb. sakei*. Furthermore, the inoculation of meat with *P. fragi* resulted in higher amounts of volatiles as compared to meat inoculated with *P. putida*. These observations strengthened the hypothesis that the volatile production is strain-dependent [16]. To sum up, the results of the present work supported the idea that microorganisms in meat perform differently when co-cultured in dual or in mixed culture species than when added as monocultures in sterile meat. It is already known that bacterial interactions play a key role in the nutrient’s contribution (which are used as principal precursors of the microbial metabolites associated to spoilage), either with synergistic/syntrophic behavior or with competitive behavior [10,12].

## 4. Conclusions

In the current study, it was found that different strains shared a different volatilome when meat was inoculated with monocultures, dual or mixed cultures. Among the detected volatile compounds, 2-butanone, diacetyl and acetaldehyde were the compounds mostly associated with the inoculated meat, while acetoin and hexanal were associated with the sterile meat. *P. fragi* exhibited the highest spoilage potential at all cases and was associated with the highest number of volatiles. In addition, *Ln. mesenteroides* shared a more abundant volatile profile in contrast to *Lb. sakei*; however, the main volatiles associated with the LAB strains were alcohols and carbonyls with seven or eight carbon atoms. Terpenoids, dimethyl sulfide and aldehydes indicated fresh meat, whereas 2-ethyl-1-hexanol and esters were more prominent in spoiled samples. However, the interaction of strains that do not contribute directly to spoilage (causing unpleasant changes) may contribute in the generation of associated spoilage compounds or interact with the produced molecules, leading to a complicated profile of sensory spoilage. The above observations could be fundamental in understanding the complexity of spoilage bacteria and their spoilage potential in terms of the produced metabolites and the possible impact on meat quality.

## Figures and Tables

**Table 1:** Estimated kinetic parameters of different microbial groups in sterile pork meat stored at 4 °C.

Inoculation	Lag Phase (h) ^1^	y_0_ (log_10_CFU/g) ^2^	y_end_ (log_10_CFU/g) ^3^	μ_max_ (h^−1^) ^4^	R^2^
**Monoculture**					
*Pseudomonas fragi* (F)	9.31 ± 10.04	1.95 ± 0.22	10.51 ± 2.38	0.04 ± 0.01	0.99
*Pseudomonas putida* (P)	148.23 ± 15.43	2.26 ± 0.21	- ^5^	0.01 ± 0.01	-
*Leuconostoc mesenteroides* (M)	30.98 ± 14.01	1.75 ± 0.20	-	0.02 ± 0.01	0.86
*Lactobacillus sakei* (S)	50.67 ± 16.45	30.98 ± 14.01	-	0.02 ± 0.00	0.96
**Dual Culture**					
F + P (CF)	7.39 ± 18.55	2.26 ± 0.32	8.86 ± 0.28	0.05 ± 0.01	0.98
M + S (MF)	50.67 ± 16.45	2.03 ± 0.19	-	0.02 ± 0.00	0.91
**Cocktail**					
CF (cocktail)	68.31 ± 17.44	2.07 ± 0.55	9.00 ± 0.57	0.04 ± 0.01	0.94
MF (cocktail)	17.80 ± 33.98	2.19 ± 0.42	6.82 ± 10.46	0.02 ± 0.01	0.84

^1^: lag phase, ^2^y_0_: initial microbial population, ^3^y_end_: final microbial population, ^4^μ_max_: maximum specific growth rate and ^5^: not determined. CF: dual cultures of *P. fragi* and *P. putida*, MF: dual cultures of *Ln*. *mesenteroides* and *Lb. sakei*. The description of coded treatments is referred to in Table S1.

**Table 2:** Estimated kinetic parameters of different microbial groups in sterile pork meat stored at 10 °C.

Inoculation	Lag Phase (h) ^1^	y_0_ (log_10_CFU/g) ^2^	y_end_ (log_10_CFU/g) ^3^	μ_max_ (h^−1^) ^4^	R^2^
**Monoculture**					
*P. fragi* (F)	1.24 ± 3.01	1.99 ± 0.14	10.18 ± 0.17	0.09 ± 0.00	1.00
*P. putida* (P)	13.87 ± 9.63	2.80 ± 0.39	9.34 ± 0.54	0.07 ± 0.01	0.95
*Ln. mesenteroides* (M)	8.82 ± 7.92	1.86 ± 0.33	7.93 ± 0.37	0.08 ± 0.01	0.96
*Lb. sakei* (S)	24.72 ± 6.32	2.02 ± 0.19	7.49 ± 0.31	0.06 ± 0.01	0.97
**Dual Culture**					
F + P (CF)	2.22 ± 3.3	2.35 ± 0.15	9.52 ± 0.14	0.08 ± 0.00	0.99
M + S (MF)	26.77 ± 6.17	2.36 ± 0.26	7.91 ± 0.31	0.10 ± 0.01	0.95
**Cocktail**					
CF (cocktail)	17.80± 5.09	1.73± 0.23	9.27± 0.29	0.09± 0.01	0.99
MF (cocktail)	10.31± 6.24	2.04± 0.22	8.28± 0.28	0.06± 0.01	0.98

^1^: lag phase, ^2^y_0_: initial microbial population, ^3^y_end_: final microbial population, and ^4^μ_max_: maximum specific growth rate. CF: dual cultures of *P. fragi* and *P. putida*, MF: dual cultures of *Ln*. *mesenteroides* and *Lb. sakei*. The description of coded treatments is referred to in Table S1.

## Supplementary Materials

The following are available online at https://www.mdpi.com/2304-8158/9/5/633/s1: Table S1: Coded names for sterile meat and the inoculated meat samples with the monocultures, dual and mixed cultures of the 4 examined strains at 4 and 10 °C. Table S2: Sensory analysis of pork meat in correlation to storage time and population level, stored aerobically at 4 and 10 °C. Table S3: Fifty-one volatile compounds identified in sterile and inoculated pork samples that were further used in statistical analysis and association with the discrimination process. Figure S1: Important features identintified by PLS-DA for sterile meat and meat inoculated with: (**a**) *P. putida* (monoculture) and (**b**) *Lb. sakei* (monoculture) stored at 4 and 10 °C. The color scale represents the scaled abundance of each variable, with red indicating high abundance and green indicating low abundance. Figure S2: Dendrogram obtained after Ward-linkage clustering based on the Euclidean correlation coefficients of the 51 volatiles identified for the sterile meat and meat inoculated with mono and dual cultures of pseudomonads. The description of the coded treatments is referred to in Table S1.
