# Supplementary material for: Spoilage Potential of Pseudomonas (P. fragi, P. putida) and LAB (Leuconostoc mesenteroides, Lactobacillus sakei) Strains and Their Volatilome Profile during Storage of Sterile Pork Meat Using GC/MS and Data Analytics
Source: Foods. 2020 May 14;9(5):633. doi: 10.3390/foods9050633 (PMC7278706; doi:10.3390/foods9050633)
Supplement: Supplementary file 1 [file foods-09-00633-s001.pdf]

Table S1. Coded names for sterile meat and the inoculated meat samples with the monocultures, dual and mixed cultures of the 4 examined strains at 4 and 10 °C.

Sample	Coded names
Sterile pork meat	Control (B)
Monocultures	*Pseudomonas fragi* (F4, F10)
	Pseudomonas putida (P4, P10)
	Lactobacillus sakei (S4, S10)
	Leuconostoc mesenteroides (M4, M10)
Dual cultures	*P. fragi* and *P. putida* (CF4, CF10)
	Lb. sakei and *Ln. mesenteroides* (MF4, MF10)
Mixed culture of 4 strains	*P. fragi* and *P. putida* and *Lb. sakei* and *Ln. mesenteroides* (C4, C10)

Table S2: Sensory analysis of pork meat in correlation to storage time and population level, stored aerobically at 4 and 10 °C.

Samples	Air 4 °C			Air 10 °C		
	Inoculum (log CFU/g)	Semi-Fresh	Spoiled	Inoculum (log CFU/g)	Semi-Fresh	Spoiled
**Monoculture**						
P. fragi (F)	2.01 [1]	4.40	7.09	2.03	5.63	8.31
Time (h)	0	54 [2]	116 [3]	0	42	72
P. putida (P)	2.33	2.16	2.58	2.57	5.20	8.40
Time (h)	0	68	140	0	42	80
Ln, mesenteroides (M)	1.76	2.54	3.36	2.14	5.36	7.62
Time (h)	0	92	124.5	0	46	93
L. sakei (S)	1.75	2.40	3.57	1.74	3.26	6.23
Time (h)	0	92	140	0	42	93
**Dual Culture**						
F + P (CF)	2.32	5.11	6.74	2.25	4.00	8.52
Time (h)	0	68	104	0	26	72
M + S (MF)	2.26	2.46	4.76	2.22	4.12	7.23
Time (h)	0	54	140	0	34	80
**Cocktail**						
CF (cocktail)	2.25	3.76	6.49	1.90	2.70	6.94
Time (h)	0	54	104	0	22	80
MF (cocktail)	2.37	2.54	3.73	2.95	3.01	6.19
Time	0	54	104	0	22	80

[1]: population below the detection limit of the method; [2]: time that the sensory panel classified the sample as Semi Fresh; [3]: time that the sensory panel classified the sample as Spoiled

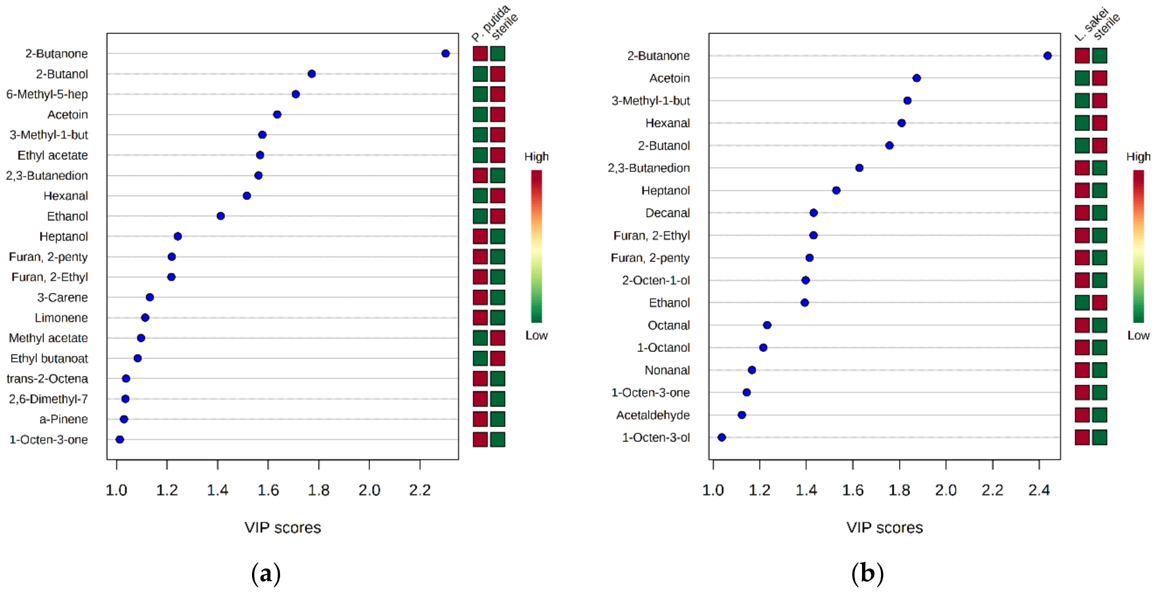

Figure S1. Important features identintified by PLS-DA for sterile meat and meat inoculated with: (**a**) *P. putida* (monoculture); (**b**) and *L. sakei* (monoculture) stored at 4 and 10 °C. The color scale represents the scaled abundance of each variable, with red indicating high abundance and green indicating low abundance.

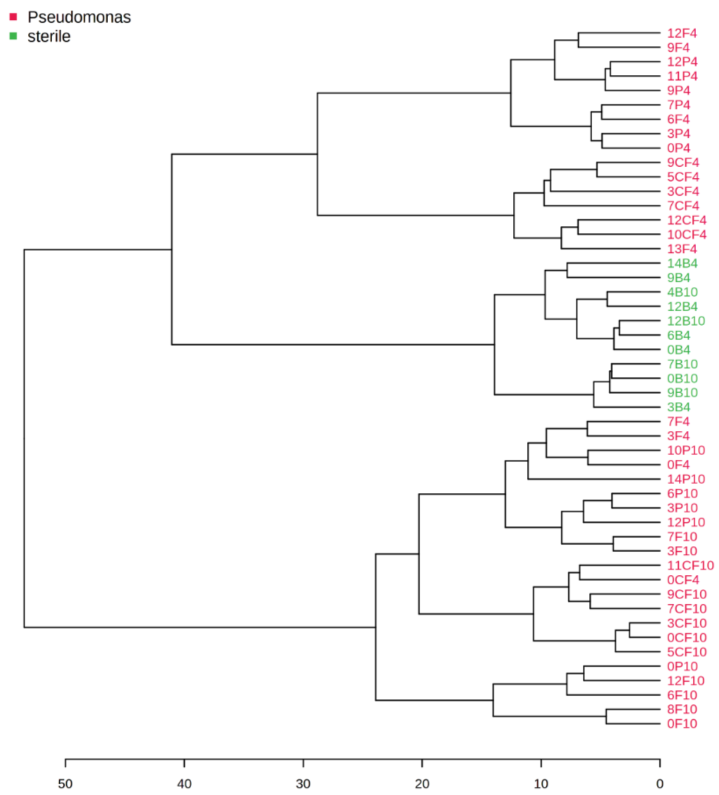

Figure S2: Dendrogram obtained after Ward-linkage clustering based on the Euclidean correlation coefficients of the 51 volatiles identified for the sterile meat and meat inoculated with mono and dual cultures of pseudomonads. The description of coded treatments is referred to Table S1.

Table S3. 51 Volatile compounds identified in sterile and inoculated pork samples that were further used in statistical analysis and association to the discrimination process.

Name	Target ion	RI_{exp} [1]	RI_{ref} [2]	Reliability of Identification[3]
Acetaldehyde	29	407	404	A
Ethanol	45	437	427	A
Dimethyl sulfide	62	520	520	B
Methyl acetate	74	527	526	A
Propanol	31	555	555	A
2,3-Butanedione	86	594	595	A
2-Butanone	72	601	598	B
2-Butanol	45	603	603	B
Ethyl acetate	61	613	606	A
1-Butanol	56	658	659	A
1-Penten-3-ol	57	675	680	A
2-Pentanone	43	686	685	B
Pentanal	58	697	699	A
2-Ethylfuran	81	702	703	B
Acetoin (3-Hydroxy-2-butanone)	88	720	713	A
3-Methyl-1-butanol	55	725	736	A
1-Pentanol	42	759	765	A
Hexanal	82	802	800	A
Ethyl butanoate	88	805	802	A
Ethyl lactate	45	815	815	A
Butyl acetate	43	818	812	A
4-methyl-1-pentanol (IS)	56	839	846	A
Hexanol	56	870	868	A
2-Heptanone	43	888	891	B
Heptanal	70	902	901	B
α-Thujene	93	926	927	A
α-Pinene	93	930	933	A
Camphene	93	944	951	B
2-Heptenal	83	954	958	B
Heptanol	70	973	970	B
Sabinene	93	973	974	B
β-Pinene	93	975	979	A
1-Octen-3-ol	57	980	980	A
1-Octen-3-one	70	980	979	B
2,5-Octanedione	43	987	984	C
6-Methyl-5-hepten-2-one	108	989	986	C
2-Pentylfuran	81	991	993	C
2-Octanone	58	993	990	C
Ethyl hexanoate	88	1001	1000	A
Octanal	43	1003	1003	A

3-Carene	93	1009	1011	B
p-Cymene	119	1024	1025	A
Limonene	93	1028	1030	A
2-Ethyl-1-hexanol	57	1030	1030	A
γ-Terpinene	93	1059	1060	A
(E)-2-Octenal	70	1060	1060	B
2-Octen-1-ol	57	1070	1066	C
1-Octanol	56	1073	1071	A
2,6-Dimethyl-7-octen-2-ol	59	1074	1081	C
2-Nonanone	58	1094	1092	C
Nonanal	57	1105	1104	B
Decanal	57	1206	1206	B

[1]RI experimental, [2]Reference from NIST14 and FFSCN, [3]A-level: agreement of retention index (RI) and mass spectrum (MS) with those of an authentic compound analyzed under identical experimental conditions; B-level: agreement of retention index (ΔRI < 20) and mass spectrum (match > 900); C-level: at least ΔRI < 20 or mass spectrum similarity match > 800
